# Diarrhea Caused by High-Fat and High-Protein Diet Was Associated With Intestinal Lactase-Producing Bacteria

**DOI:** 10.5152/tjg.2023.22451

**Published:** 2023-07-01

**Authors:** Kang Zhou, Maijiao Peng, Zhoujin Tan, Nenqun Xiao

**Affiliations:** 1 Hunan University of Chinese Medicine Faculty of Pharmacy, Changsha, Hunan, China; 2 Hunan University of Chinese Medicine Faculty of Medicine, Changsha, China

**Keywords:** High-fat and high-protein diet, diarrhea, lactase gene, lactase-producing bacteria, intestinal contents

## Abstract

**Background/Aims:**

This study aimed to investigate the effect of diarrhea induced by a high-fat and high-protein diet on lactase-producing bacteria in the intestinal contents of mice from the perspective of diarrhea-related genes.

**Materials and Methods:**

Ten specific pathogen-free Kunming male mice were chosen and randomly divided into the normal group and model group. The mice in the normal group were fed with high-fat and high-protein diet plus gavage of vegetable oil, while those in the model group were fed with general diet plus gavage of distilled water. After successful modeling, the distribution and diversity of lactase-producing bacteria in the intestinal contents were characterized by metagenomic sequencing technology.

**Results:**

After high-fat and high-protein diet intervention, Chao1, observed species index, and operational taxonomic units number decreased in the model group (*P *> .05), while the Shannon, Simpson, Pielou’s evenness, and Goods coverage indices increased (*P *> .05). The principal coordinate analysis showed that the composition of lactase-producing bacteria differed between the normal group and model group (*P *< .05). The lactase-producing bacterial source in the intestinal contents of mice was Actinobacteria, Firmicutes, and Proteobacteria, of which Actinobacteria was the most abundant phylum. At the genus level, both groups had their unique genera, respectively. Compared to the normal group, the abundance of *Bifidobacterium*, *Rhizobium,* and *Sphingobium* increased, while *Lachnoclostridium*, *Lactobacillus*, *Saccharopolyspora*, and *Sinorhizobium* decreased in the model group.

**Conclusion:**

High-fat and high-protein diet altered the structure of lactase-producing bacteria in the intestinal contents, elevating the abundance of dominant lactase-producing bacteria, while decreasing the richness of lactase-producing bacteria, which may further induce the occurrence of diarrhea.

**Main Points**

- We investigated the distribution and diversity of lactase-producing bacteria in intestinal contents of mice with diarrhea induced by high-fat and high-protein diet.
- High-fat and high-protein diet mainly alters the composition structure of the lactase-producing bacteria.
- High-fat and high-protein diet further increased the abundance of dominant lactase-producing bacteria in the intestine.

## INTRODUCTION

The intestinal microbiota is a dynamic and complex ecosystem composed of diverse microbial communities, which play an irreplaceable role in maintaining human health homeostasis.^1^ Through its rich content of carbohydrate-active enzyme genes, the intestinal microbiota can form complementarity with the host, and hence participate in the food metabolic cycle of the organism.^2^ Meanwhile, some components that are difficult to be used by the organism (e.g., dietary fiber) can be utilized by the intestinal microbiota, and partially converted into substances beneficial to the host.^3^

With the accelerated pace of modern life and the rise of living standards, highly processed foods are increasingly becoming the choice of most people. These foods are often rich in fat and protein but little in dietary fiber.^3^ However, excessive fat and protein are not conducive to the balance of absorption of substances in the body, which can easily lead to eating disorders that cause disorders in the intestinal microbiota, thus inducing diarrhea.^4-6^ And this may also be related to the lack or low activity of digestive enzymes. In the pig breeding industry, insufficient secretion of digestive enzymes or high protein and fat content in feeds can easily lead to nutritional diarrhea in weaned piglets, and enzyme supplementation or the use of enzyme preparations can reduce the occurrence of diarrhea in piglets.^7-9^ Furthermore, our previous study found that the activity of several enzymes, including lactase, was significantly decreased in the diarrhea mouse model made by high-fat and high-protein diet (HFHPD) intervention.^9^

Lactase, also known as β-galactosidase, can degrade lactose into galactose and glucose. If lactase deficiency or activity is reduced, it can lead to the symptoms of diarrhea, gastrointestinal distention, and abdominal pain.^10^ Studies have shown that various diarrheal diseases are closely related to lactase activity, including diarrhea-predominant irritable bowel syndrome, persistent diarrhea, and antibiotics-associated diarrhea (AAD).^11-14^ Meanwhile, lactase has been linked to digestive disorders. For example, Chumpitazi et al^15^ found that half of 129 pediatric patients with functional dyspepsia had disaccharidase deficiency, with lactase as the main deficiency. The intestinal microbiota is one of the important sources of the body’s lactase. And compared to animal and plant sources, microbial sources tend to have higher lactase yields.^16^ This is also evidenced by the fact that diarrhea caused by lactase deficiency can be relieved by supplementation with lactase-producing bacteria, such as *Bifidobacterium* sp.^17,18^ Moreover, due to differences in gene coding, there is variability in the activity of lactase from different strain sources, and even some lactase does not express activity.^19^ Previous studies have shown that the alleviating effect of the probiotic *Debaryomyces hansenii* on AAD is related to the modulation of key lactase-producing bacteria.^20,21^ Therefore, we speculated that on the basis of reducing the lactase activity, HFHPD might also affect specific lactase-producing bacteria, causing damage to the microbial enzyme metabolic pathway. Simultaneously, the genetic diversity of enzymes can well represent the differences between microbial ecological groups, which has good prospects for application in environmental pollution, antibiotic resistance, and atmospheric circulation.^13,22,23^ However, the relationship between an unhealthy diet (especially HPHPD) and related functional enzyme genes of microbiota is not clear.

From the perspective of diarrhea-related microbial enzyme genes, we investigated the diversity and distribution characteristics of the lactase-producing bacterial in the intestinal contents of mice with diarrhea caused by HFHPD, aiming to reveal the mechanism of diarrhea caused by HFHPD, and provide an experimental basis for the development of new strategies and new potential targets of drugs for diarrhea treatment caused by improper diet.

## MATERIALS AND METHODS

### Animals

Ten specific pathogen-free Kunming male mice weighing 18-22 g were purchased from Hunan Sleika Jingda Experimental Animal Co. Ltd [SCXK (Xiang) 2019-0004] and housed in the Animal Experiment Center of Hunan University of Chinese Medicine (room temperature 23°C-25°C, relative humidity 50-70%). The process of animal experiments was conducted under animal protocols approved by Animal Ethics and Welfare Committee of the Hunan University of Chinese Medicine.

### Diets

The general feed was purchased from Hunan Sleika Jingda Experimental Animal Co (protein: 20%, fat: 4%, and lactose: 0%). High-fat and high-protein feed was made by mixing milk powder (Nestle, protein: 30%, lactose: 0%, and fat: 20%; Harbin, Heilongjiang Province, China), soybean milk flour (Huiyi, protein: 33%, lactose: 0%, and fat: 18%; Shantou, Guangdong Province, China), flour (Huiyi, protein: 13%, lactose: 0%, and fat: 2%) and meat pine (AnhuiLizheng, 30% protein, lactose: 0%, and 25% fat, Hefei, Anhui Province, China) in the ratio of 1:2:2:1. Vegetable oil (Arawan, lactose: 0%, Shanghai, China).

### Reagents

Proteinase K, lysozyme, Tris-saturated phenol: chloroform: isoamyl alcohol (25:24:1), TE buffer, and acetone were purchased from Beijing Dingguo Biotechnology Co. Ltd. About 0.1 mol/L phosphate-buffered solution (PBS) buffer, TE buffer, 10% sodium dodecyl sulfate (SDS), 5 mol/L NaCl, hexadecyl trimethyl ammonium bromide (CTAB)/NaCl, chloroform: isoamyl alcohol (24:1), 3 mol/L sodium acetate and 70% anhydrous ethanol, were prepared in the laboratory.

### Methods

**Figure 1.**
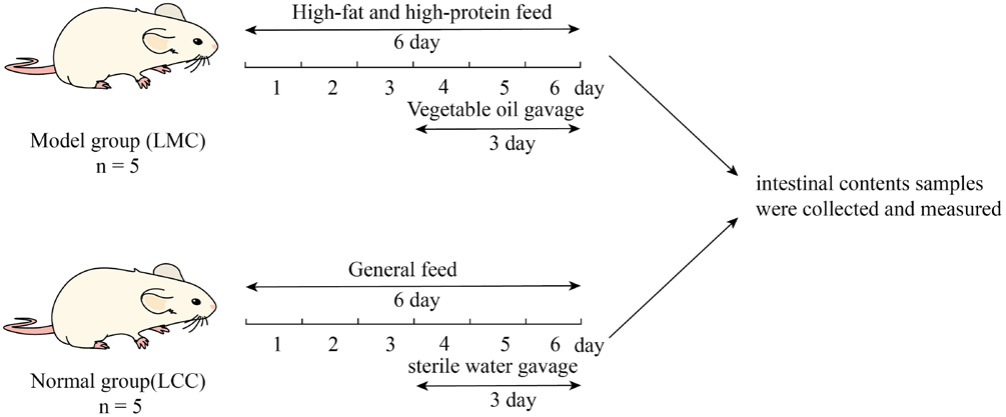
Experimental design.

Ten mice were fed adaptively for 2 days and then randomly divided into normal (LCC) and model groups (LMC) with 5 mice in each group.^9^ Mice in the LMC were fed with HFHPD and gavage with vegetable oil (0.4 mL/time, twice a day for 3 days) on day 4. Mice in the LMC were fed with general feed and gavage with equal amounts of sterile water. The modeling phase lasted 6 days, and the mice were considered to be successfully modeled when diarrhea occurred. The experimental design is shown in Figure 1.

### Sampling of Intestinal Contents from Mice

At the end of the modeling, the mice were sacrificed by cervical dislocation, and the intestinal tract of the mice was cut open under sterile conditions. The contents from the jejunum to ileum were taken with sterile forceps, placed in a centrifuge tube, marked and weighed, and stored at −20°C for subsequent use.^24^

### DNA Extraction

2.0 g of the collected intestinal contents were placed in a 50 mL sterile centrifuge tube, homogenized in 30 mL of 0.1 mol/L PBS, and centrifuged at 200 × g for 2 min. The supernatant was transferred to a new sterile tube after being washed twice with PBS, centrifuged at 10 000 × g for 8 min, and the precipitate was collected after being washed twice with PBS and once with acetone. Phosphate-buffered solution was washed 3 times before being resuspended in 4 mL TE buffer. According to our earlier report,^25^ following sample pretreatment, high-quality DNA was obtained by lysozyme wall breaking, proteinase K lysis, SDS lysis, CTAB treatment, and phenol/chloroform extraction.

### Polymerase Chain Reaction Amplification and Sequencing

Lactase gene amplification was performed using the universal primers reported by the group previously,^13,26,27^ upstream primer: 5’-TRRGCAACGAATACGGSTG-3’ and downstream primer: 5’-ACCATGAARTTSGTGGTSARCGG-3’. Polymerase chain reaction (PCR) amplification system: Q5 high-fidelity DNA polymerase 0.25 μL, 5 × reaction buffer 5 μL, 5 × high GC buffer 5 μL, deoxy-ribonucleoside triphosphate (dNTP) (10 mM) 0.5 μL, template DNA 1 μL, upstream primer (10 μM) 1 μL, downstream primer (10 μM) 1 μL and ultrapure water 11.25 μL. Polymerase chain reaction amplification conditions: 98°C for 30 s, then perform 98°C for 15 s, 46°C for 30 s, 72°C for 30 s, for a total of 32 cycles, extend for 5 min after 72°C, and store at 4°C. After the PCR products were quantified and quality-checked, the samples were sequenced according to the standard procedure of the Illumina Miseq platform. The sequencing work was performed by Shanghai Personalbio Technology Co.

### Bioinformatics Analysis

The obtained sequences were divided into operational taxonomic units (OTUs)^28^ using Qiime2 (2019.4, http://qiime.org/)^29^ with a 97% similarity threshold, and matched with the NCBI database for species annotation analysis. Alpha diversity was expressed by Chao.^30^ Observed species, Shannon,^31^ Simpson,^32^ Pielou’s evenness,^33^ and Goods coverage^34^ indices. Unweighted pair-group method with arithmetic means (UPGMA) clustering tree, principal coordinate analysis (PCoA), and Adonis difference analysis were used to represent beta diversity analysis of lactase-producing bacteria, and marker difference species were searched for by linear discriminant analysis effect size (LEfSe).

### Statistical Analysis

The data were analyzed using The Statistical Package for Social Sciences version 25.0 software (IBM Corp.; Armonk, NY, USA) and the data were expressed as mean ± SD (*x* ± *s*). Data were analyzed by independent sample *t*-test or Wilcoxon rank-sum test according to whether the data were normally distributed and the variance was uniform, and *P *< .05 in the analysis of variance was considered significant.

## RESULTS

### Behavioral Observation and Fecal Water Content in Mice

**Figure 2.**
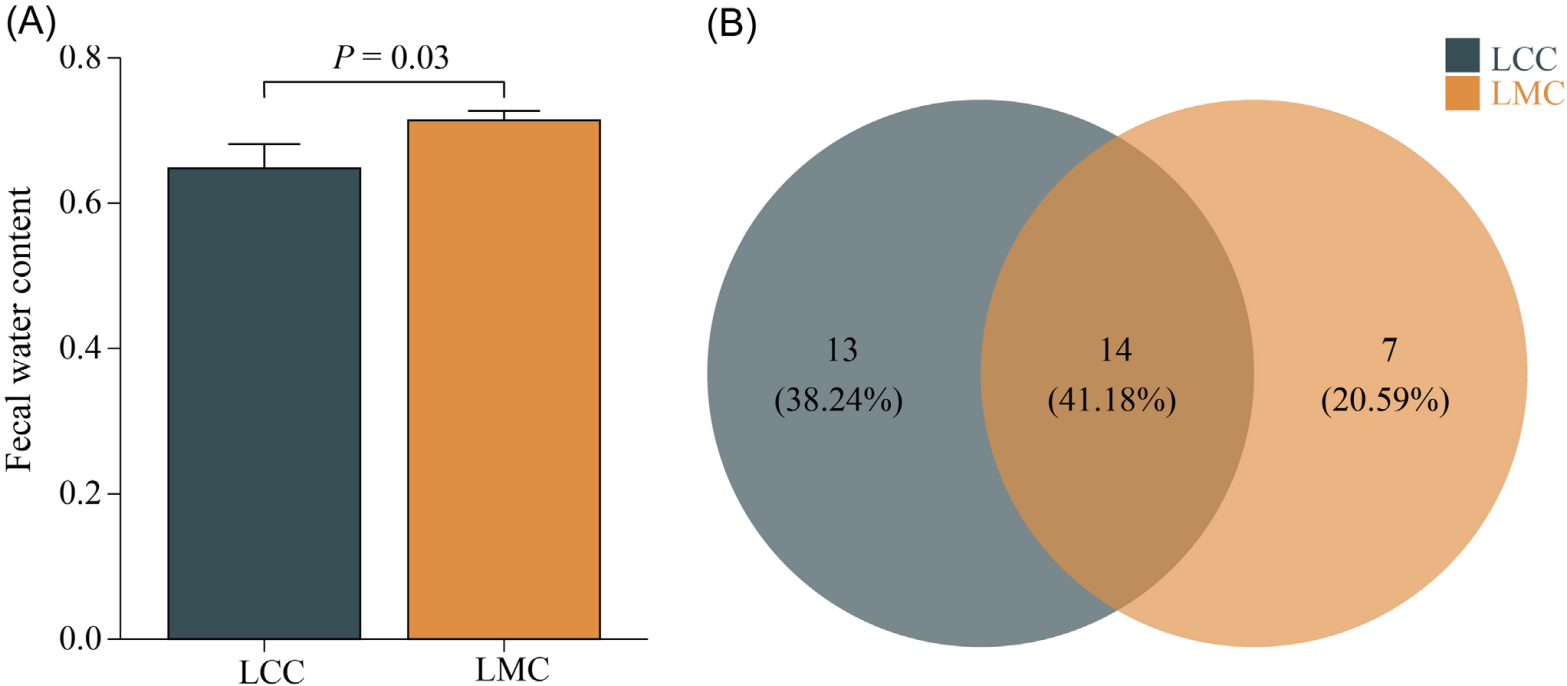
(A) Fecal water content. (B) Venn diagram of OTU distribution of lactase-producing bacteria in intestinal contents. LCC, normal group; LMC, model group; OTU, operational taxonomic unit.

After 6 days of HFHPD, the LCC had black feces with a slightly hard texture and shiny fur. In the LMC, the feces were brownish-yellow in texture and thin and wet; more than half of them became paste-like, sticky, and scattered at the tail and anus. Meanwhile, we examined the fecal water content (Figure 2A) and found that the fecal water content of LMC mice was significantly higher than that of LCC mice (71.43% vs. 65.83%, *P *< .05).

### Effect of High-Fat and High-Protein Diet on Lactase-Producing Bacterial Operational Taxonomic Unit in Intestinal Contents

**Figure 3.**
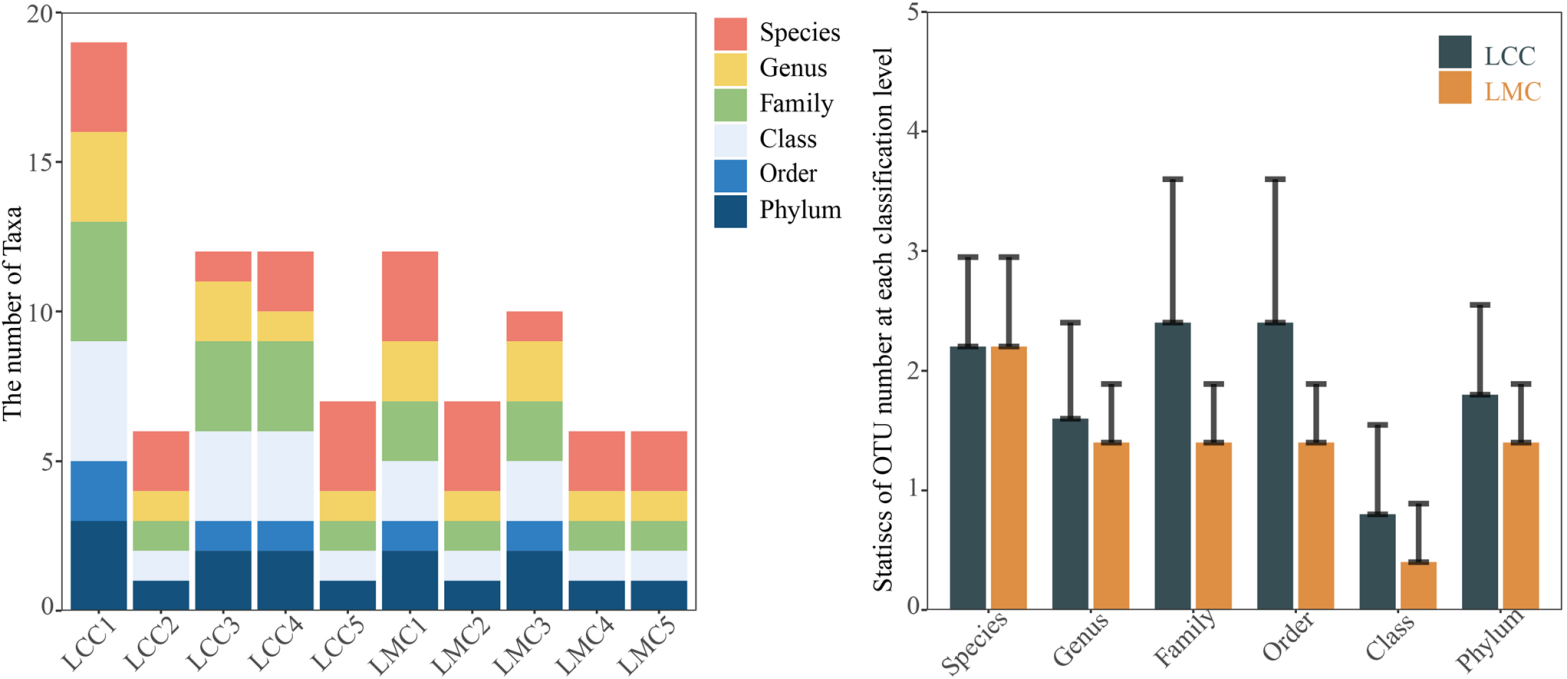
Number and differences of lactase-producing bacterial OTUs in mouse intestinal contents at different taxonomic levels. Data were expressed as (mean ± SD). LCC, normal group; LMC, model group; OTUs, operational taxonomic units.

Venn diagram depicts the numbers of OTUs exclusive to or shared between distinct groups. As seen in Figure 2B, the number of OTUs unique to LCC is 13, or 38.24%; the number of OTUs unique to LMC is 7, or 20.59%; the number of OTUs common to both groups was 14, or 41.18%. And the overall trend of the number of OTUs in the LCC was higher than that in the LMC at each classification level (Figure 3). It is suggested that HFHPD reduced the number of lactase-producing bacteria taxonomic units in the intestinal contents of mice.

### Alpha Diversity Analysis

**Figure 4.**
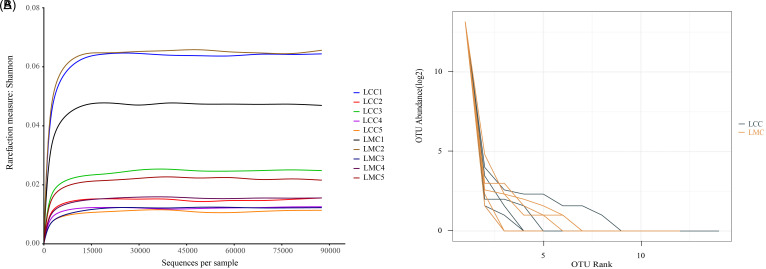
(A) Rarefaction curve. The curve’s flatness reflects the effect of sequencing depth on the diversity of the observed samples; the flatter the curve, the more the sequencing results are sufficient to reflect the diversity contained in the current samples. (B) Rank abundance curve. The length of the folded line on the horizontal axis reflects the number of OTUs in that sample at that abundance. The fold line’s flatness reflects the community composition’s evenness; the flatter the fold line, the smaller the difference in abundance among OTUs in the community. LCC, normal group; LMC, model group; OTUs, operational taxonomic units.

Rarefaction curves can evaluate the diversity of each sample with increasing sequencing depth. As seen in Figure 4A, the increase in diversity has flattened out, indicating that the current sequencing depth is sufficient for further analysis. Rank abundance curves can reflect the distribution pattern of species in the samples. In Figure 4B, we can see that the broken lines of the LMC and the LCC are mixed and both are steeper, indicating that the OTU abundance in both the LMC and the LCC are more different and less evenness, suggesting that both groups have some kind of dominant bacteria.

**Figure 5.**
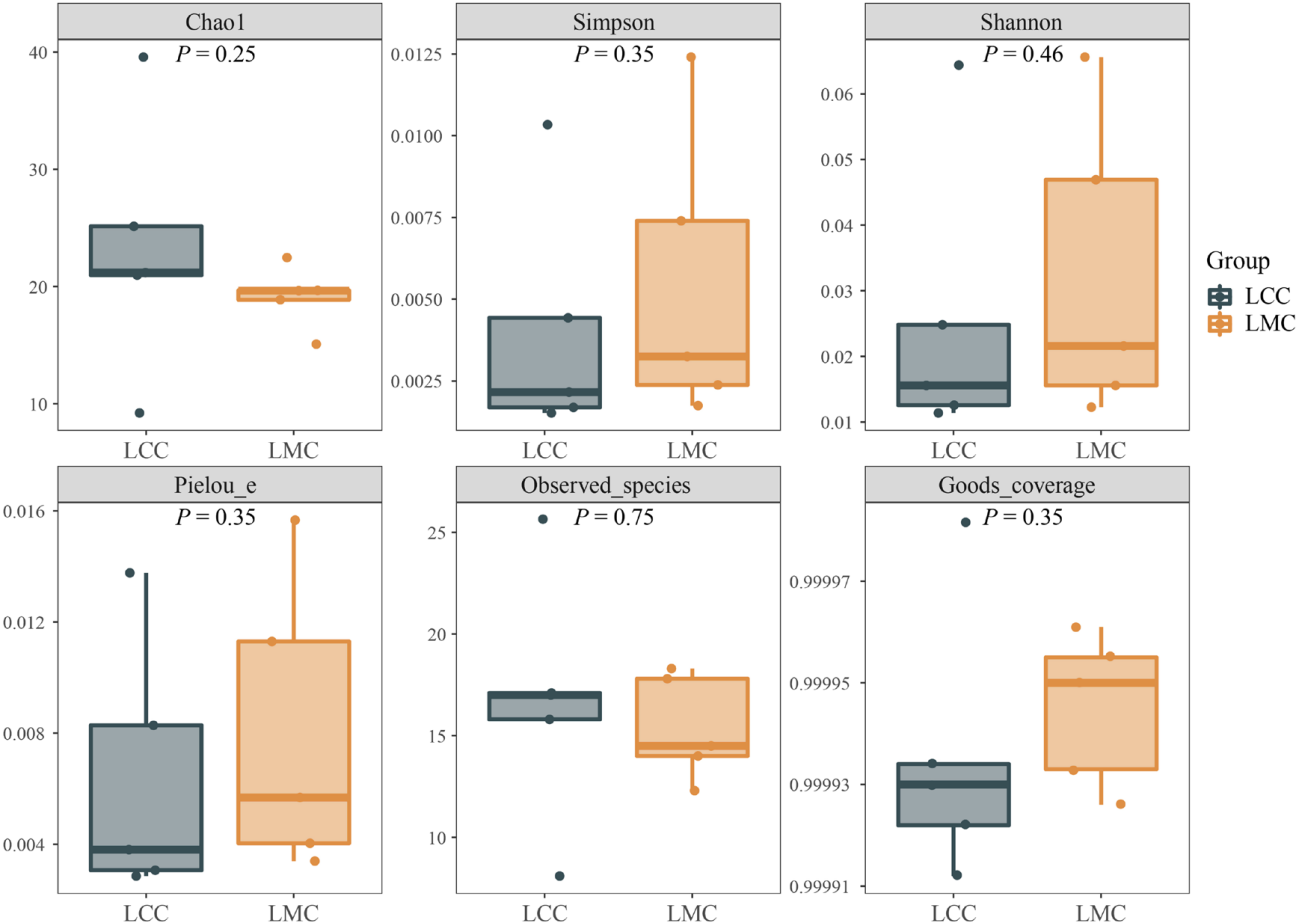
Alpha diversity index of lactase-producing bacteria in the intestinal contents of high-fat and high-protein diet mice. The numbers under the diversity index label are the *P* values of the Wilcoxon rank-sum test or independent sample *t*-test. LCC, normal group; LMC, model group.

Alpha diversity values are represented by richness (Chao^30^ and observed species index), diversity (Shannon^31^ and Simpson^32^ index), evenness (Pielou’s^33^ evenness index), and species coverage (Good’s^34^ coverage index). As seen in Figure 5, the Good’s^34^ coverage index of both groups is close to 1, indicating that the current sample has very few undetected species. Compared with the LCC, Shannon^31^, Simpson^32^, Pielou’s^33^ evenness and Good’s^34^ coverage indices increased, while Chao^30^ and observed species indices decreased in the LMC, but none of them were significantly different (*P *> .05). It is suggested that HFHPD decreased the richness of lactase-producing bacteria in the intestinal contents while increasing their diversity.

### Beta Diversity Analysis

**Figure 6.**
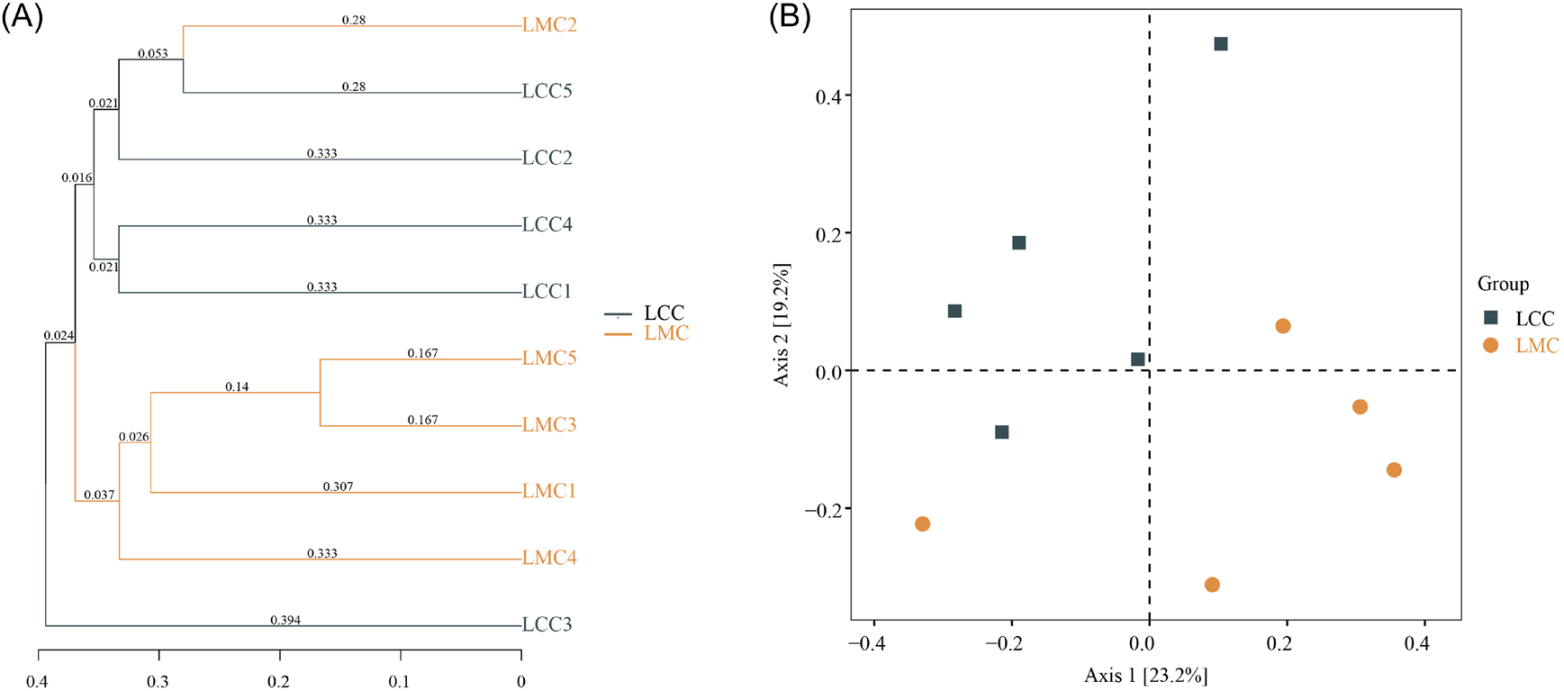
(A) Unweighted pair-group method with arithmetic means clustering tree based on Jaccard distances. (B) Principal coordinate analysis based on Jaccard distances. The closer the distance between the 2 points, the more similar the microbial community structure in the 2 samples. LCC, normal group; LMC, model group.

The similarity of community structure across samples can be examined using beta diversity analysis. In UPGMA (Figure 6A), except for LCC3 and LMC2, the LCC and LMC were well separated at 0.024 and clustered in 2 separate groups. The main distribution characteristics of the samples can be obtained by dimensionality reduction of the microbiota data by PCoA, thus quantifying the differences and similarities of the lactase-producing bacteria in different intestinal contents samples.^35^ In PCoA (Figure 6B), the first and second principal components of 23.2% and 19.2%, respectively, and the LCC and LMC can be well separated, while the sample distribution of both groups was relatively scattered. The LCC samples were mainly clustered in the second quadrant, while the LMC was mainly distributed in the fourth quadrant, and the Adonis test showed significant structural differences between the 2 groups (*P *< .05). Overall, the UPGMA and PCoA suggested that HFHPD altered the structure of lactase-producing bacteria in the intestinal contents.

### Distribution Composition of Lactase-Producing Bacteria in Intestinal Contents

**Figure 7.**
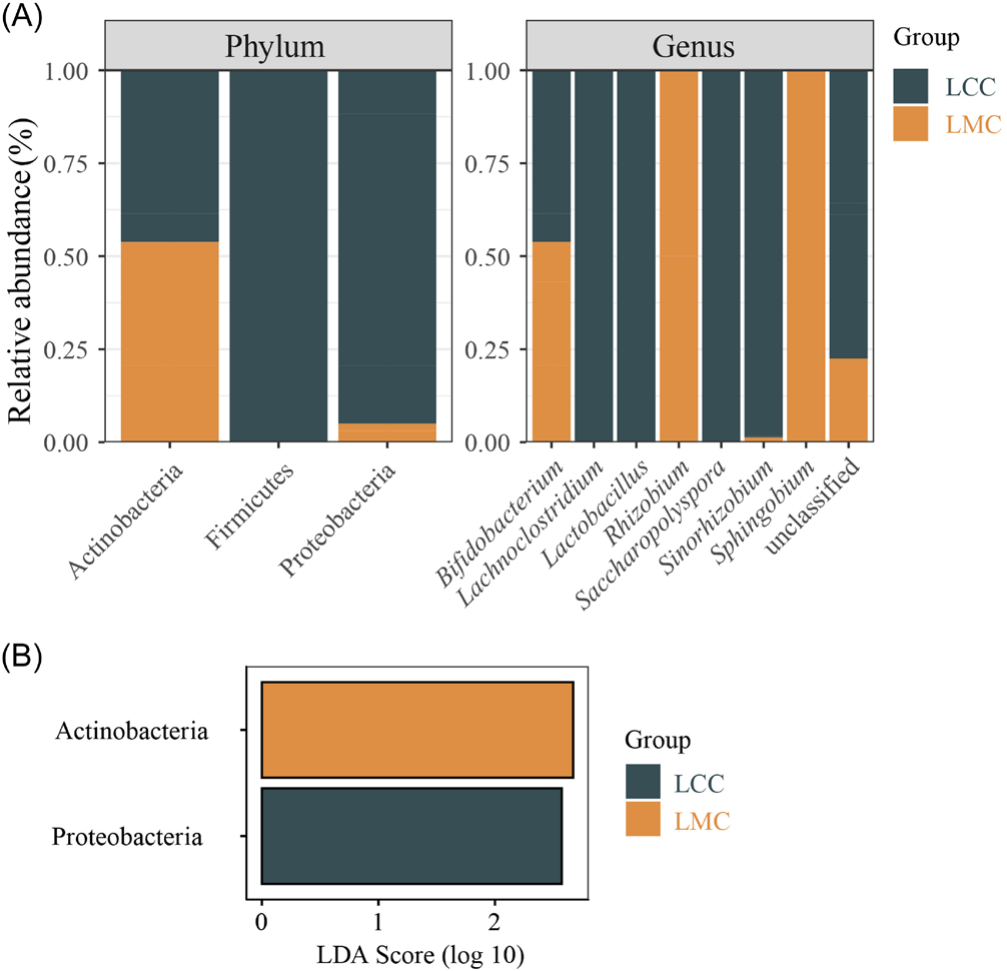
(A) Percentage of LCC and LMC in different taxonomic compositions. (B) Histogram of the marker species LEfSe (Linear discriminant analysis [LDA] score >2). LCC, normal group; LefSe, linear discriminant analysis effect size; LMC, model group.

The distribution of lactase-producing bacteria in intestinal contents was obtained according to the taxonomic identification of OTU, and the results are shown in Table 1 and Figure 7A. From Table 1, the lactase-producing bacteria sources were Actinobacteria, Firmicutes, and Proteobacteria, with Actinobacteria being the dominant phylum, with more than 99% abundance in both groups, and Firmicutes being exclusive to the LCC. The lactase-producing bacteria sources at the genus level were *Bifidobacterium*, *Lachnoclostridium*, *Lactobacillus*, *Rhizobium*, *Saccharopolyspora*,* Sinorhizobium*, *Sphingobium,* and unclassified, where *Bifidobacterium* was the dominant genus with more than 99% abundance in both groups, *Saccharopolyspora*, *Lachnoclostridium*, and *Lactobacillus* were exclusive to the LCC, and *Rhizobium* and *Sphingobium* were exclusive to the LMC. As seen from Figure 7A, compared to the LCC, the abundance of Actinobacteria increased, while the abundance of Firmicutes and Proteobacteria decreased in the LMC. At the genus level, the abundance of *Bifidobacterium*,* Rhizobium*, and *Sphingobium* increased, while the abundance of *Lachnoclostridium*, *Lactobacillus*, *Saccharopolyspora,* and *Sinorhizobium* decreased in the LMC.

### Linear Discriminant Analysis Effect Size of Lactase-Producing Bacteria From Intestinal Contents

To reveal the characteristic lactase-producing bacterial taxa with significant differences between the LCC and LMC, we performed the LEfSe. As seen in Figure 7B, the marker difference species for the LMC was Actinobacteria and for the LCC was Proteobacteria.

## Discussion

Diet is the primary factor influencing intestinal microbiota, and studies have shown that over 50% of microbiota structural variation in mice and over 20% in humans are related to diet.^36,37^ Among them, high-fat diet can trigger systemic inflammation and imbalance of intestinal homeostasis by modulating inflammation-associated bacterial strains, increasing intestinal mucosal permeability, and promoting the secretion of inflammatory factors and translocation of bacterial lipopolysaccharides.^38,39^ While moderate protein improves health, excessive high-protein diets can still hurt intestinal homeostasis. Unlike fats and carbohydrates, intestinal microbial fermentation is the main metabolic pathway for proteins. And toxic products (amines, H_2_S, and ammonia) from excess protein fermentation can decrease short-chain fatty acid yields, increase paracellular permeability and alter epithelial cell morphology, thereby compromise intestinal health.^40,41^

Diet quality is usually positively correlated with intestinal microbiota diversity.^42^ Interestingly, the HFHPD intervention increased the diversity of lactase-producing bacteria in this study, but the richness was decreased. This may be related to specific dietary differences or environments. For example, studies have shown that a high-fat diet can reduce microbiota diversity,^43^ but Wang et al^44^ found that other components mixed in the high-fat diet can increase microbiota diversity. Snelson et al^45^ reported that long-term high-protein diet mainly changed the microbiota structure, but had little correlation with microbiota diversity. Meanwhile, in the taxonomic composition (Table 1), there is a clear dominant lactase-producing bacteria (*Bifidobacterium*) in the intestinal content of mice. Compared to LCC, LMC was missing the lactase-producing bacteria *Lactobacillus*,* Lachnoclostridium*, and *Saccharopolyspora*. Among them, *Lactobacillus* is one of the most widely used lactase-producing bacteria with functions such as inhibition of intestinal pathogenic bacteria, promotion of helper T-cell development, induction of cytokine production, and enhancement of cellular immune function.^46^ Moreover, the structure of the lactase-producing bacterial composition was significantly altered by HFHPD intervention, and both LMC and LCC had their exclusive genera. The secretion of differentially active enzymes to regulate host interaction with ingested substances is an important means by which the intestinal microbiota affects host health.^47^ For example, Li et al^48^ reported that *Streptococcus thermophilus* downregulates Hippo pathway kinases by secreting lactase, which inhibits tumorigenesis. Thus, changes in the structure of lactase-producing bacteria may mean a decrease in the intestinal microbiota’s ability to regulate the host’s physiological state.

The LEfSe results suggest that the increase in the abundance of Actinobacteria, especially the representative *Bifidobacterium*, is a key change in lactase-producing bacteria caused by HFHPD. Notably, *Bifidobacterium* is recognized as a probiotic that can affect the metabolism of lipids with anti-inflammatory properties by altering the associated microbiota.^49^ And both *Bifidobacterium longum* and *Bifidobacterium animals *can be used to alleviate lactose intolerance.^50^ But Brandao et al^51^ found a positive correlation between the abundance of *Bifidobacteria* and both dairy intake and diarrhea in lactose intolerant patients. Lactose is artificially converted to lactulose and used as a laxative. A review study also mentioned that low doses of lactulose significantly increased the number of *Bifidobacterium* and *Lactobacillus* spp.^52^ This indicated that diarrhea induced by HFHPD had a certain correlation with *Bifidobacterium*. Nevertheless, an increase in the dominant lactase-producing bacteria does not imply an elevation in lactase activity. Protein activity is closely related to its structure and modifications.^53^ Therefore, the normal expression of lactase genes may also be hindered by the HFHPD-altered intestinal environment, such as pH,^54^ which affects lactase activity in the intestine.

Finally, there are still some limitations in this study, that is, the activity of microbial lactase gene expression was not verified. Simultaneously, due to the variability of microbial ecological regions, different regions of the intestinal microbiota reflect different responses to dietary factors. In this study, we only preliminarily investigated the characteristics of the bacterial lactase gene in intestinal contents under the intervention of HFHPD, and other intestinal regions still need to be further explored.

## Figures and Tables

**Table 1.** Composition of Lactase-Producing Bacteria of Intestinal Contents at the Phylum and Genus Levels

	LCC	LMC
Actinobacteria	0.999169 ± 0.000871	0.999972 ± 0.000033
Firmicutes	0.000268 ± 0.000522	0.000000 ± 0.000000
Proteobacteria	0.000563 ± 0.000698	0.000028 ± 0.000033
*Bifidobacterium*	0.998964 ± 0.001104	0.999908 ± 0.000049
*Saccharopolyspora*	0.000091 ± 0.000182	0.000000 ± 0.000000
*Lactobacillus*	0.000262 ± 0.000525	0.000000 ± 0.000000
*Lachnoclostridium*	0.000006 ± 0.000012	0.000000 ± 0.000000
*Rhizobium*	0.000000 ± 0.000000	0.000003 ± 0.000004
*Sinorhizobium*	0.000389 ± 0.000745	0.000004 ± 0.000006
*Sphingobium*	0.000000 ± 0.000000	0.000018 ± 0.000035
Unclassified	0.000288 ± 0.000288	0.000067 ± 0.000052

Data are expressed as mean ± SD.

LCC, normal group; LMC, model group.

## References

[1] Needham BD Funabashi M Adame MD et al. A gut-derived metabolite alters brain activity and anxiety behaviour in mice. Nature. 2022;602(7898):647 653. (10.1038/s41586-022-04396-8) 35165440 PMC9170029

[2] El Kaoutari A Armougom F Gordon JI Raoult D Henrissat B . The abundance and variety of carbohydrate-active enzymes in the human gut microbiota. Nat Rev Microbiol. 2013;11(7):497 504. (10.1038/nrmicro3050) 23748339

[3] Barber TM Kabisch S Pfeiffer AFH Weickert MO . The health benefits of dietary fibre. Nutrients. 2020;12(10):3209. (10.3390/nu12103209) PMC7589116 33096647

[4] Huang P Liu Y . A Reasonable Diet promotes balance of intestinal microbiota: prevention of precolorectal cancer. BioMed Res Int. 2019;2019:3405278. (10.1155/2019/3405278) PMC6683831 31428633

[5] Zhu JY Zheng T Liu YW Peng MJ Tan ZJ . Changes of intestinal mucosal bacteria after diarrhea in mice induced by high-fat and high-protein diet. J Food Nutr Res. 2022;10(2):88 97. (10.12691/jfnr-10-2-2)

[6] Schulz MD Atay C Heringer J et al. High-fat-diet-mediated dysbiosis promotes intestinal carcinogenesis independently of obesity. Nature. 2014;514(7523):508 512. (10.1038/nature13398) 25174708 PMC4233209

[7] Gao J Yin J Xu K Li T Yin Y . What is the impact of diet on nutritional diarrhea associated with gut microbiota in weaning piglets: a system review. BioMed Res Int. 2019;2019:6916189. (10.1155/2019/6916189) PMC6949732 31976326

[8] Tian M Chen J Wu Z et al. Fat encapsulation reduces diarrhea in piglets partially by repairing the intestinal barrier and improving fatty acid Transport. Animals (Basel). 2020;11(1):28. (10.3390/ani11010028) PMC7824132 33375218

[9] Shao HQ He YS Xiao NQ Xie GZ Tan ZJ . Establishment of a mouse model of diarrhea with gastrointestinal food stagnation syndrome and the efficacy of Baohe wan. Shih-chen Kuo I Kuo Yao. 2022;33:10 15.

[10] Goh LH Mohd Said R Goh KL . Lactase deficiency and lactose intolerance in a multiracial Asian population in Malaysia. JGH Open. 2018;2(6):307 310. (10.1002/jgh3.12089) 30619942 PMC6308090

[11] Parfenov AI Akhmadullina OV Sabelnikova EA et al. Disaccharidase deficiency and functional bowel diseases. Ter Arkh. 2017;89(4):45 52. (10.17116/terarkh201789445-52) 28514399

[12] Long CX He L Guo YF Liu YW Xiao NQ Tan ZJ . Diversity of bacterial lactase genes in intestinal contents of mice with antibiotics-induced diarrhea. World J Gastroenterol. 2017;23(42):7584 7593. (10.3748/wjg.v23.i42.7584) 29204058 PMC5698251

[13] Long CX Liu YW He L et al. Bacterial lactase genes diversity in intestinal mucosa of mice with dysbacterial diarrhea induced by antibiotics. 3 Biotech. 2018;8(3):176. (10.1007/s13205-018-1191-5) PMC5847641 29556430

[14] Xue H Zhang M Ma J Chen T Wang F Tang X . Lactose-induced chronic diarrhea results from abnormal luminal microbial fermentation and disorder of ion transport in the colon. Front Physiol. 2020;11:877. (10.3389/fphys.2020.00877) PMC7403511 32848839

[15] Chumpitazi BP Robayo-Torres CC Tsai CM et al. Demographic and clinical correlates of mucosal disaccharidase deficiencies in children with functional dyspepsia. J Pediatr Gastroenterol Nutr. 2018;66(suppl 3):S52 S55. (10.1097/MPG.0000000000001859) 29762379 PMC5957288

[16] Saqib S Akram A Halim SA Tassaduq R . Sources of β-galactosidase and its applications in food industry. 3 Biotech. 2017;7(1):79. (10.1007/s13205-017-0645-5) PMC5429307 28500401

[17] Popović N Brdarić E Đokić J et al. Yogurt produced by novel natural starter cultures improves gut epithelial barrier in vitro. Microorganisms. 2020;8(10):1586. (10.3390/microorganisms8101586) PMC7602395 33076224

[18] Masoumi SJ Mehrabani D Saberifiroozi M Fattahi MR Moradi F Najafi M . The effect of yogurt fortified with *Lactobacillus acidophilus* and *Bifidobacterium* *sp.* probiotic in patients with lactose intolerance. Food Sci Nutr. 2021;9(3):1704 1711. (10.1002/fsn3.2145) 33747481 PMC7958570

[19] Aburto C Castillo C Cornejo F et al. β-galactosidase from Exiguobacterium acetylicum: cloning, expression, purification and characterization. Bioresour Technol. 2019;277:211 215. (10.1016/j.biortech.2019.01.005) 30639092

[20] He YS Tang Y Peng MJ Xie GZ Li WG Tan ZJ . Influence of *Debaryomyces hansenii* on bacterial lactase gene diversity in intestinal mucosa of mice with antibiotic-associated diarrhea. PLoS One. 2019;14(12):e0225802. (10.1371/journal.pone.0225802) PMC6897403 31809511

[21] Wu Y Tang Y Xiao NQ Wang CH Tan ZJ . Bacterial lactase gene characteristics in intestinal contents of antibiotic-associated diarrhea mice treated with *Debaryomyces hansenii* . Med Sci Monit. 2020;26:e920879. (10.12659/MSM.920879) PMC7003665 31986127

[22] Escalas A Hale L Voordeckers JW et al. Microbial functional diversity: from concepts to applications. Ecol Evol. 2019;9(20):12000 12016. (10.1002/ece3.5670) 31695904 PMC6822047

[23] Ramírez-Flandes S González B Ulloa O . Redox traits characterize the organization of global microbial communities. Proc Natl Acad Sci U S A. 2019;116(9):3630 3635. (10.1073/pnas.1817554116) 30808753 PMC6397516

[24] Shao HQ Zhang CY Xiao NQ Tan ZJ . Gut microbiota characteristics in mice with antibiotic-associated diarrhea. BMC Microbiol. 2020;20(1):313. (10.1186/s12866-020-01999-x) PMC7559773 33059603

[25] Hui HY Wu Y Zheng T Zhou SN Tan ZJ . Bacterial characteristics in intestinal contents of antibiotic-associated diarrhea mice treated with Qiweibaizhu powder. Med Sci Monit. 2020;26:e921771. (10.12659/MSM.921771) PMC7245059 32398636

[26] He L Liu YW Guo YF Shen KJ Hui HY Tan ZJ . Diversity of intestinal bacterial lactase gene in antibiotics-induced diarrhea mice treated with Chinese herbs compound Qi Wei Bai Zhu San. 3 Biotech. 2018;8(1):4. (10.1007/s13205-017-1024-y) PMC5718989 29242764

[27] Long CX He L Liu YJ et al. Universal primer for analysis of the diversity of intestinal bacterial lactase gene. Chin J Appl Environ Biol. 2017;23(4):758 763.

[28] Blaxter M Mann J Chapman T et al. Defining operational taxonomic units using DNA barcode data. Philos Trans R Soc Lond B Biol Sci. 2005;360(1462):1935 1943. (10.1098/rstb.2005.1725) 16214751 PMC1609233

[29] Caporaso JG Kuczynski J Stombaugh J et al. QIIME allows analysis of high-throughput community sequencing data. Nat Methods. 2010;7(5):335 336. (10.1038/nmeth.f.303) 20383131 PMC3156573

[30] Chao A Nonparametric estimation of the number of classes in a population. Scand J Stat. 1984;11:265 270.

[31] Shannon CE A mathematical theory of communication. Bell Syst Tech J. 1948;27(3):379 423. (10.1002/j.1538-7305.1948.tb01338.x)

[32] Simpson EH Measurement of diversity. Nature. 1949;163(4148):688 688. (10.1038/163688a0)

[33] Pielou EC The measurement of diversity in different types of biological collections. J Theor Biol. 1966;13:131 144. (10.1016/0022-5193(66)90013-0)

[34] Good IJ The population frequencies of species and the estimation of population parameters. Biometrika. 1953;40(3-4):237 264. (10.1093/biomet/40.3-4.237)

[35] Ramette A Multivariate analyses in microbial ecology. FEMS Microbiol Ecol. 2007;62(2):142 160. (10.1111/j.1574-6941.2007.00375.x) 17892477 PMC2121141

[36] Leeming ER Johnson AJ Spector TD Le Roy CI . Effect of diet on the gut microbiota: rethinking intervention duration. Nutrients. 2019;11(12):2862. (10.3390/nu11122862) PMC6950569 31766592

[37] Rothschild D Weissbrod O Barkan E et al. Environment dominates over host genetics in shaping human gut microbiota. Nature. 2018;555(7695):210 215. (10.1038/nature25973) 29489753

[38] Luo Q Cheng D Huang C et al. Improvement of colonic immune function with soy isoflavones in high-fat diet-induced obese rats. Molecules. 2019;24(6):1139. (10.3390/molecules24061139) PMC6470843 30909396

[39] Li XY Peng XX Guo KX Tan ZJ . Bacterial diversity in intestinal mucosa of mice fed with Dendrobium officinale and high-fat diet. 3 Biotech. 2021;11(1):22. (10.1007/s13205-020-02558-x) PMC7779387 33442520

[40] Cai J Chen Z Wu W Lin Q Liang Y . High animal protein diet and gut microbiota in human health. Crit Rev Food Sci Nutr. 2021:1 14. 10.1080/10408398.2021.1898336 33724115

[41] Mefferd CC Bhute SS Phan JR et al. A high-fat/high-protein, Atkins-type diet exacerbates *Clostridioides (clostridium)* *difficile* infection in mice, whereas a high-carbohydrate diet protects. mSystems. 2020;5(1):e00765-19. (10.1128/mSystems.00765-19) PMC7018531 32047064

[42] Laitinen K Mokkala K . Overall dietary quality relates to gut microbiota diversity and abundance. Int J Mol Sci. 2019;20(8):1835. (10.3390/ijms20081835) PMC6515207 31013927

[43] Zhang C Zhang M Pang X Zhao Y Wang L Zhao L . Structural resilience of the gut microbiota in adult mice under high-fat dietary perturbations. ISME J. 2012;6(10):1848 1857. (10.1038/ismej.2012.27) 22495068 PMC3446802

[44] Wang BT Kong QM Li X et al. A high-fat diet increases gut microbiota biodiversity and energy expenditure due to nutrient difference. Nutrients. 2020;12(10):3197. (10.3390/nu12103197) PMC7589760 33092019

[45] Snelson M Clarke RE Nguyen TV et al. Long term high protein diet feeding alters the microbiome and increases intestinal permeability, systemic inflammation and kidney injury in mice. Mol Nutr Food Res. 2021;65(8):e2000851. (10.1002/mnfr.202000851) 33547877

[46] Abraham N Namachivayam C Sundaramoorthy S . Lactobacillus- an friendly bacteria. Int J Technol. 2021:70 77. (10.52711/2231-3915.2021.00010)

[47] Onyango SO Juma J De Paepe K Van de Wiele T . Oral and gut microbial carbohydrate-active enzymes landscape in health and disease. Front Microbiol. 2021;12:653448. (10.3389/fmicb.2021.653448) PMC8702856 34956106

[48] Li Q Hu W Liu WX et al. *Streptococcus thermophilus* inhibits colorectal tumorigenesis through secreting β-galactosidase. Gastroenterology. 2021;160(4):1179 1193.e14. (10.1053/j.gastro.2020.09.003) 32920015

[49] Yamamura R Okubo R Katsumata N et al. Lipid and energy metabolism of the gut microbiota is associated with the response to probiotic Bifidobacterium breve strain for anxiety and depressive symptoms in schizophrenia. J Pers Med. 2021;11(10):987. (10.3390/jpm11100987) PMC8539730 34683128

[50] Oak SJ Jha R . The effects of probiotics in lactose intolerance: a systematic review. Crit Rev Food Sci Nutr. 2019;59(11):1675 1683. (10.1080/10408398.2018.1425977) 29425071

[51] Brandao Gois MF Sinha T Spreckels JE et al. Role of the gut microbiome in mediating lactose intolerance symptoms. Gut. 2022;71(1):215 217. (10.1136/gutjnl-2020-323911) 34086598 PMC8666824

[52] Karakan T Tuohy KM Janssen-van Solingen G . Low-dose lactulose as a prebiotic for improved gut health and enhanced mineral absorption. Front Nutr. 2021;8:672925. (10.3389/fnut.2021.672925) PMC8353095 34386514

[53] Zhong WQ Song QF Cheng T Li J . Research on lactase and its gene engineering. zhongguo rupin gongye. 2008;3:47 50.

[54] Bibbò S Ianiro G Giorgio V et al. The role of diet on gut microbiota composition. Eur Rev Med Pharmacol Sci. 2016;20(22):4742 4749. 27906427

